# Goose Deoxycholic Acid Ameliorates Liver Injury in Laying Hens with Fatty Liver Hemorrhage Syndrome by Inhibiting the Inflammatory Response

**DOI:** 10.3390/ijms26010429

**Published:** 2025-01-06

**Authors:** Nannan Wang, Weiwei Li, Guangyi Ouyang, Hengqi Li, Jiancheng Yang, Gaofeng Wu

**Affiliations:** Liaoning Provincial Key Laboratory of Zoonosis, College of Animal Science and Veterinary Medicine, Shenyang Agricultural University, Shenyang 110866, China; 2021200169@stu.syau.edu.cn (N.W.);

**Keywords:** goose deoxycholic acid, M1-type polarization, M2-type polarization, liver damage, FLHS laying hens

## Abstract

Fatty liver hemorrhagic syndrome (FLHS) in laying hens is a nutritional and metabolic disease involving liver enlargement, hepatic steatosis, and hepatic hemorrhage as the primary symptoms. The syndrome is prone to occur during the peak laying period of laying hens, which has resulted in significant economic losses in the laying hen breeding industry; however, the specific pathogenesis of FLHS remains unclear. Our group and previous studies have shown that bile acid levels are significantly decreased during the development of fatty liver and that targeted activation of bile acid–related signaling pathways is beneficial for preventing and treating fatty liver. In this study, we generated a FLHS laying hen model by feeding hens a high-energy, low-protein diet, with goose deoxycholic acid (CDCA) given as an intervention. HE staining, fluorescence quantitative PCR, and ELISA were used to evaluate the effects of CDCA on pathological changes and inflammatory responses in the liver. The results showed that hepatic hemorrhage in FLHS laying hens was reduced after CDCA treatment. Furthermore, fat vacuoles and transaminase levels decreased significantly. In addition, expression levels of M1-type macrophage markers and polarization products were significantly reduced, and the expression of pro-inflammatory regulatory factors related to the JAK-STAT signaling pathway, LPS-TLR4-Myd88–NF-kB signaling pathway, and NLRP3 inflammasomes decreased significantly as well. Expression levels of M2-type macrophage markers and polarization products increased significantly, as did the expression of anti-inflammatory regulators related to the JAK-STAT signaling pathway. These results suggest that CDCA ameliorates liver injury in laying hens with FLHS by inhibiting macrophage M1-type polarization and the resulting pro-inflammatory response, thereby promoting M2-type macrophage polarization and an anti-inflammatory response.

## 1. Introduction

Fatty liver hemorrhagic syndrome (FLHS) in laying hens, discovered and named by Couch in 1956, is a nutritional metabolic disease characterized by hepatic hemorrhage and hepatic steatosis [1]. The causes include genetic, nutritional, environmental, and other factors, such as management practices. The disease is prone to occur in caged laying hens in good condition at the peak of laying, and the onset of the disease can lead to a decline in egg production. Under modern intensive farming conditions, the pathogenesis of FLHS is primarily related to high feed energy intake. Although the specific pathogenesis remains unclear, the pathological mechanism is similar to that of non-alcoholic fatty liver disease. The “second strike” hypothesis proposed by Day et al. [2] in 1998 is the currently accepted rational explanation for non-alcoholic fatty liver disease (NAFLD). This hypothesis suggests that the first strike is excessive accumulation of triglycerides and free fatty acids in the liver due to factors such as excessive lipid intake, which results in hepatic steatosis. The lack of viability of steatotic hepatocytes then triggers an oxidative stress response. The second strike involves the multiple impacts of oxidative stress, mitochondrial damage, and the inflammatory response in the liver, all of which exacerbate the effects of the first strike, thus further aggravating liver injury.

The hepatic inflammatory response is primarily mediated by macrophages in the liver. Macrophages, when activated, differentiate into M1 type and M2 type. Recent studies have shown that stimulation of the TLR4 receptor on the surface of liver macrophages by LPS leads to the polarization of macrophages toward the M1 type, which secrete a variety of pro-inflammatory factors, primarily including interleukin (IL)-1, tumor necrosis factor–alpha (TNF-alpha), and oxygen free radicals, which can lead to inflammation-related injury in the liver [3]. The classical theory suggests that LPS stimulation causes inflammatory injury in the liver, and during the development of hepatic inflammation, binding of IL-4 to macrophage receptors activates JAK, promotes STAT phosphorylation and macrophage polarization toward the M2 type, inhibits the pro-inflammatory response, and ameliorates inflammatory injury in the liver [4]. M2-type macrophages secrete active substances such as IL-4 and IL-10 to alleviate the inflammatory response and simultaneously induce apoptosis of M1-type macrophages to attenuate inflammatory injury in the liver.

Many recent studies have confirmed that the physiological effects of bile acids synthesized by the liver have an important impact on lipid metabolism, glucose metabolism, inflammation, and other functions through activation of the farnesol X receptor and G protein–coupled receptor [5]. Although the study by Oleszycka Ewa et al. emphasized the role of bile acids at physiological doses in stimulating the production of pro-inflammatory factors and recruiting innate immune cells in the pathogenesis of inflammatory diseases [6], many recent studies have demonstrated that artificially added bile acids can be effective in reducing inflammation in disease states. Preliminary studies by our group have shown that the bile acid profile changes significantly during FLHS; CDCA levels in particular are significantly reduced. Thus, prophylactic application of CDCA can inhibit the occurrence of FLHS. It has been reported that bile acid membrane receptor G protein–coupled receptor 5 (TGR5) is expressed primarily on the surface membrane of macrophages, and CDCA is, in turn, a major agonist of TGR5. Other studies have shown that bile acid binding to TGR5 results in significant anti-inflammatory effects, whereas other studies reported that CDCA exerts anti-inflammatory effects by activating the TGR5 signaling pathway [7,8]. We therefore hypothesized that CDCA treatment could ameliorate liver injury by modulating macrophage-mediated inflammatory responses in the liver of laying hens with FLHS.

In this study, we generated a model of FLHS in laying hens by feeding hens high-energy/low-protein diets and intervened with the addition of CDCA to evaluate its effect on liver injury, pro-inflammatory responses mediated by M1-type macrophage polarization, and anti-inflammatory responses mediated by M2-type macrophage polarization. The ultimate objective of the study was to elucidate the regulatory effect of CDCA on inflammation-related injury in the liver of laying hens with FLHS and the possible mechanism, thereby providing a theoretical basis for the application of CDCA in the prevention and treatment of FLHS in laying hens.

## 2. Results

### 2.1. CDCA Ameliorates Effects of Liver Injury

**Figure 1:**
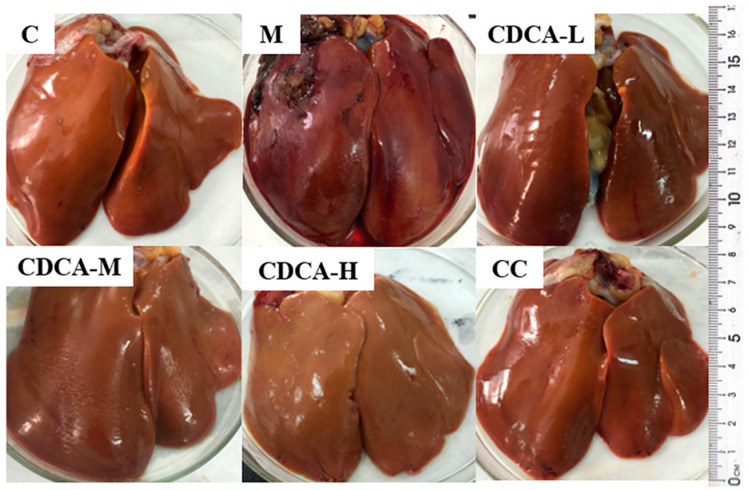
Morphology of the liver of laying hens.

Figure 1 shows a histological image of liver tissue from a test hen. The livers of laying hens in Group C exhibited a smooth and shiny surface, firm texture, and normal, dark-red color without signs of hemorrhage, whereas the livers of laying hens in Group M showed an earthy-yellow color, soft texture, crumbled easily when pinched with fingers, and exhibited large areas of hemorrhaging or blood clots on the surface. Hepatic surface hemorrhaging in laying hens was effectively alleviated by CDCA intervention, similar to the results observed with Group C. One of the criteria for diagnosing FLHS is the presence of a large number of hemorrhages or blood clots on the surface of the liver. The success of the FLHS model development could be judged based on observations of the liver during necropsy. The addition of CDCA to the diet effectively reduced the occurrence of FLHS.

**Figure 2:**
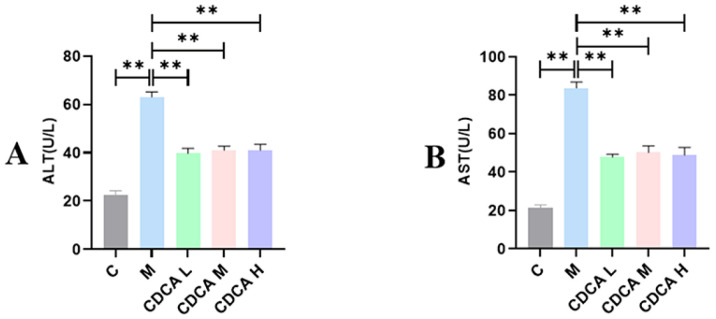
Liver function indices in laying hens. (**A**) Effect of CDCA addition on serum ALT levels in FLHS laying hens. (**B**) Effect of CDCA addition on serum AST levels in FLHS laying hens. Data are expressed as mean ± SEM. ** *p* < 0.01.

Serum levels of ALT and AST in test laying hens were significantly higher in the FLHS model group compared with the normal group (Figure 2; *p* < 0.05), and the addition of CDCA lowered the levels even further (*p* < 0.01).

**Figure 3:**
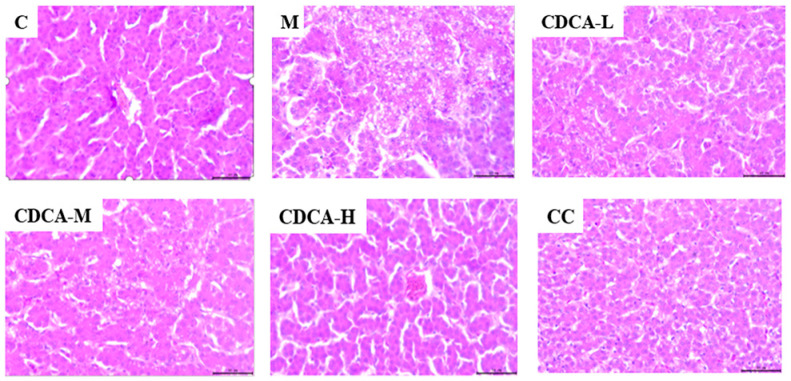
Histological analysis of liver tissues of laying hens (HE 400×).

The results of HE staining of liver sections from test laying hens are shown in Figure 3. In the normal control group, hepatocyte cords were clearly visible, and no fat droplets were observed. Compared with the control group, some of the hepatocyte cord structures in liver sections from the FLHS group had disappeared, and typical manifestations of hepatic steatosis were evident. Furthermore, fat vacuoles appeared large in both size and number, and based on this evidence, it was judged that development of the FLHS model was successful. Fatty vacuoles in hepatocytes were smaller in size and fewer in number after CDCA treatment, representing significant signs of recovery relative to Group M.

### 2.2. CDCA Inhibits Macrophage M1-Type Polarization-Mediated Pro-Inflammatory Responses

**Figure 4:**
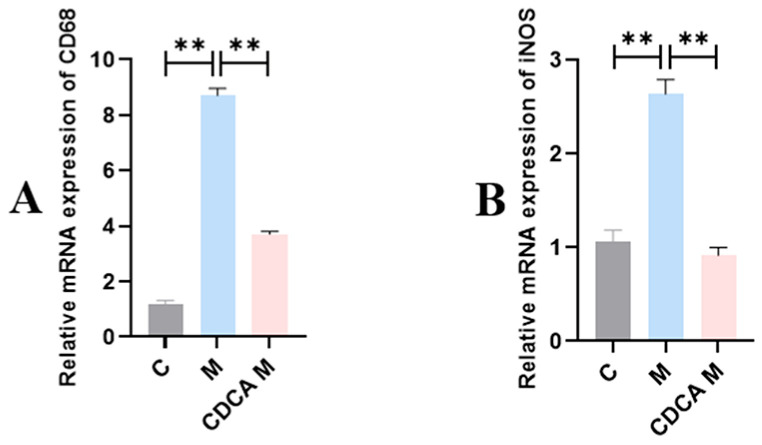
Analysis of liver macrophage markers in laying hens. (**A**) Effect of CDCA on CD68 mRNA expression in FLHS laying hens. (**B**) Effect of CDCA on iNOS mRNA expression in FLHS laying hens. Data are shown as mean ± SEM. ** *p* < 0.01.

The results of liver M1 macrophage marker assay in laying hens are shown in Figure 4. The expression levels of mRNAs encoding CD68 and iNOS were significantly elevated in the FLHS model group compared with the normal control group (*p* < 0.01). Expression levels of CD68 and iNOS mRNAs were significantly reduced compared with the control group following CDCA treatment (*p* < 0.01).

**Figure 5:**
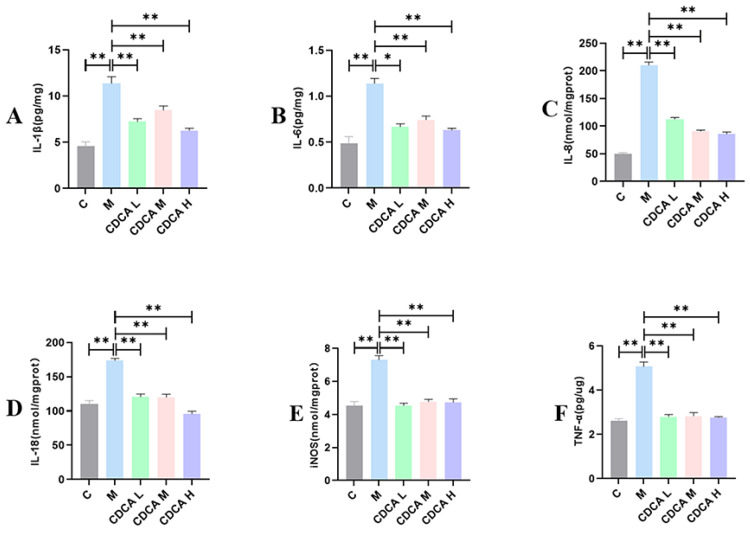
Analysis of liver pro-inflammatory factors in laying hens. (**A**) Effect of CDCA on he patic IL-1β levels in FLHS laying hens. (**B**) Effect of CDCA on hepatic IL-6 levels in FLHS laying hens. (**C**) Effect of CDCA on hepatic IL-8 levels in FLHS laying hens. (**D**) Effect of CDCA on hepatic IL-18 levels in FLHS laying hens. (**E**) Effect of CDCA on hepatic iNOS levels in FLHS laying hens. (**F**) Effect of CDCA on hepatic TNF-α levels in FLHS laying hens. Data are expressed as mean ± SEM. * *p* < 0.05 and ** *p* < 0.01.

Relative expression levels of M1-type macrophage–associated factors are shown in Figure 5. The levels of IL-1β, IL-6, IL-8, IL-18, iNOS, and TNF-α were highly significantly elevated in the FLHS model group compared with the normal control group (*p* < 0.01). Levels of these markers were significantly reduced compared with the control group following CDCA treatment (*p* < 0.01).

**Figure 6:**
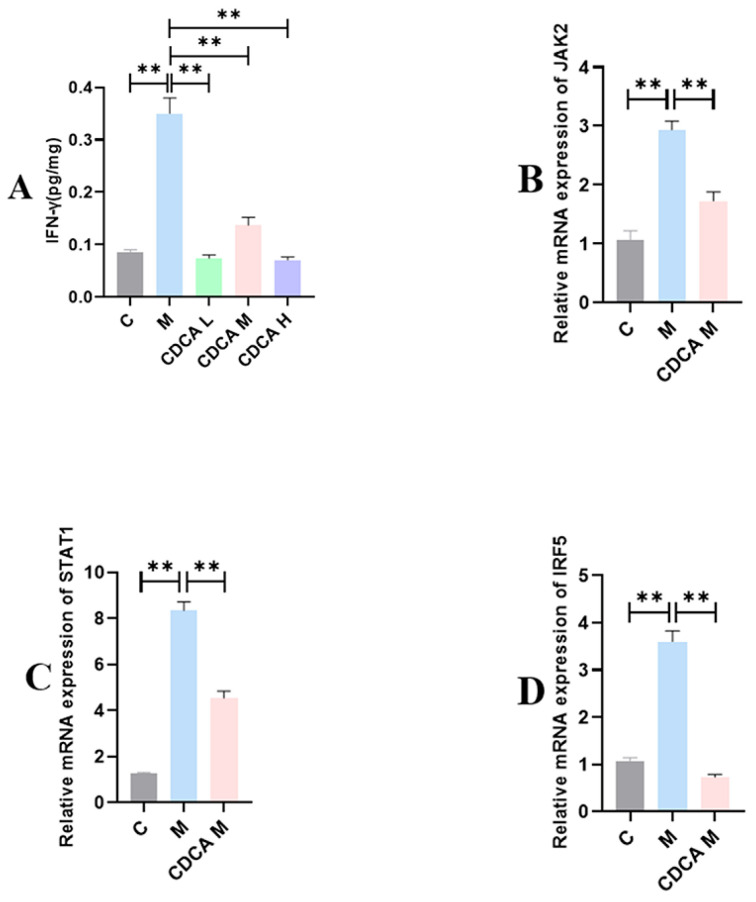
Analysis of M1-type macrophage-mediated signaling pathway-associated factors. (**A**) Effect of CDCA on hepatic IFN-γ levels in FLHS laying hens. (**B**) Effect of CDCA on hepatic JAK2 mRNA expression in FLHS laying hens. (**C**) Effect of CDCA on hepatic STAT1 mRNA expression in FLHS laying hens. (**D**) Effect of CDCA on hepatic IRF5 mRNA expression in FLHS laying hens. Data are expressed as mean ± SEM. ** *p* < 0.01.

The results of analyses of factors associated with M1-type macrophage–mediated signaling are shown in Figure 6. The expression levels of mRNAs encoding JAK2, STAT1, and IRF5, regulators of the signaling pathway activated by IFN-γ, were significantly elevated in the FLHS model group compared with the normal control group (*p* < 0.01). CDCA treatment significantly reduced the expression of IFN-γ (*p* < 0.01) as well as mRNAs encoding JAK2, STAT1, and IRF5 (*p* < 0.01).

**Figure 7:**
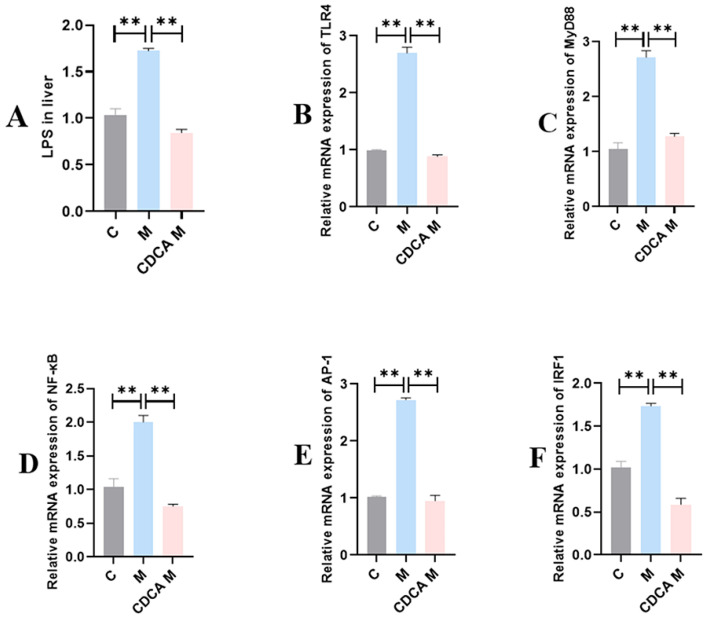
Analysis of the LPS-TLR4-MyD88-NF-κB inflammatory signaling pathway. (**A**) Effect of CDCA on LPS content in FLHS laying hens. (**B**) Effect of CDCA on TLR4 mRNA expression in FLHS laying hens. (**C**) Effect of CDCA on MyD88 mRNA expression in FLHS laying hens. (**D**) Effect of CDCA on NF-κB mRNA expression in FLHS laying hens. (**E**) Effect of CDCA on AP-1 mRNA expression in FLHS laying hens. (**F**) Effect of CDCA on IRF1 mRNA expression in FLHS laying hens. Data are expressed as mean ± SEM. ** *p* < 0.01.

The results of analyses of the LPS-TLR4-MyD88–NF-κB inflammatory signaling pathway are shown in Figure 7. Compared with the normal group, the LPS content was significantly elevated in the FLHS model group (*p* < 0.01), and the expression levels of TLR4, MyD88, NF-κB, AP-1, and IRF1 mRNAs were also significantly increased (*p* < 0.01). Following CDCA treatment, the LPS content decreased significantly (*p* < 0.01), as did the levels of mRNAs encoding TLR4, MyD88, NF-κB, AP-1, and IRF1 (*p* < 0.01).

**Figure 8:**
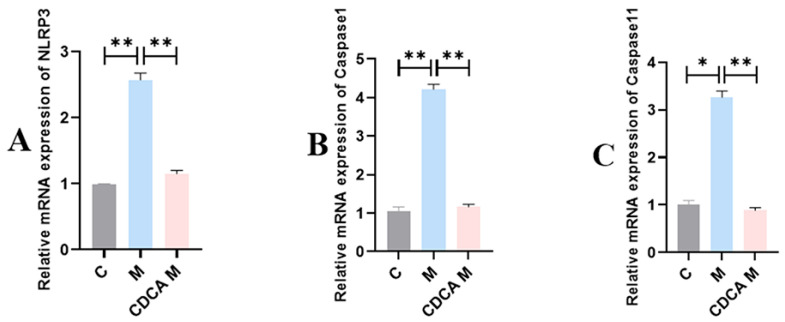
Analysis of NLRP3 inflammatory vesicle-associated factors. (**A**) Effect of CDCA on NLRP3 mRNA expression in FLHS laying hens. (**B**) Effect of CDCA on Caspase1 mRNA expression in FLHS laying hens. (**C**) Effect of CDCA on Caspase11 mRNA expression in FLHS laying hens. Data are expressed as mean ± SEM. * *p* < 0.05 and ** *p* < 0.01.

The results of analyses of NLRP3 inflammatory vesicle–associated factors are shown in Figure 8. The expression levels of mRNAs encoding NLRP3, Caspase1, and Caspase11 were significantly elevated in the FLHS model group compared with the normal control group (*p* < 0.01). Following CDCA treatment, the expression levels of mRNAs encoding NLRP3, Caspase1, and Caspase11 were significantly reduced compared with the control group (*p* < 0.01).

### 2.3. CDCA Promotes Macrophage M2-Type Polarization–Mediated Anti-Inflammatory Responses

**Figure 9:**
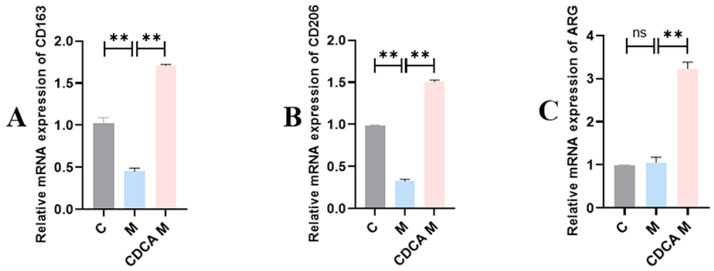
Analysis of liver macrophage markers in laying hens. (**A**) Effect of CDCA on CD163 mRNA expression in FLHS hens. (**B**) Effect of CDCA on CD206 mRNA expression in FLHS hens. (**C**) Effect of CDCA on ARG mRNA expression in FLHS hens. Data are expressed as mean ± SEM. ** *p* < 0.01, and ns (*p* > 0.05).

The results of M2 macrophage marker assays are shown in Figure 9. Compared with the normal control group, the expression levels of mRNAs encoding CD206 and CD163 were significantly reduced in the FLHS model group (*p* < 0.01), but there was no significant difference in the expression level of ARG mRNA. Following CDCA treatment, expression levels of mRNAs encoding CD206, CD163, and ARG increased significantly compared with the control group (*p* < 0.01).

**Figure 10:**
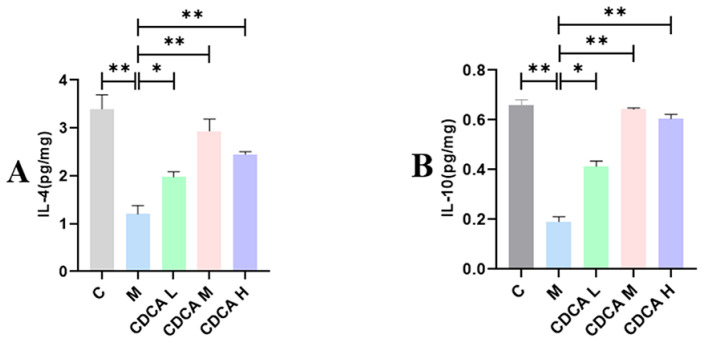
Analysis of liver anti-inflammatory factors in laying hens. (**A**) Effect of CDCA on liver IL-4 levels in FLHS laying hens. (**B**) Effect of CDCA on liver IL-10 levels in FLHS laying hens. Data are expressed as mean ± SEM. * *p* < 0.05 and ** *p* < 0.01.

The results of analyses of the expression of M2-type macrophage-associated factors are shown in Figure 10. The levels of IL-4 and IL-10 were significantly lower in the FLHS model group compared with the normal control group (*p* < 0.01). Following CDCA treatment, however, the levels of IL-4 and IL-10 increased significantly compared with the control group (*p* < 0.01).

**Figure 11:**
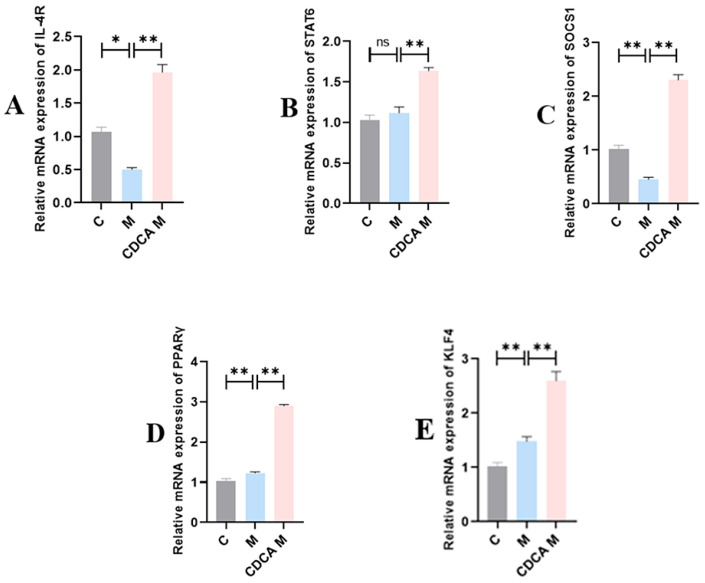
Analyses of regulators of the M2-type macrophage-mediated signaling pathway. (**A**) Effect of CDCA on hepatic IL-4R mRNA levels in FLHS laying hens. (**B**) Effect of CDCA on hepatic STAT6 mRNA expression in FLHS laying hens. (**C**) Effect of CDCA on hepatic SOCS1 mRNA expression in FLHS laying hens. (**D**) Effect of CDCA on hepatic PPARγ mRNA expression in FLHS laying hens. (**E**) Effect of CDCA on hepatic KLF4 mRNA expression in FLHS laying hens. Data are expressed as mean ± SEM. * *p* < 0.05, ** *p* < 0.01, and ns (*p* > 0.05).

Figure 11 shows the results of analyses of various M2-type macrophage-mediated signaling pathway regulators. Compared with the normal control group, the expression levels of IL-4R and SOCS1 mRNAs were significantly reduced in the FLHS model group (*p* < 0.05). By contrast, the expression of PPAR-γ and KLF4 mRNAs was significantly elevated compared with the control group (*p* < 0.05), but there was no significant difference in the expression of STAT6 mRNA. Following CDCA treatment, the expression levels of mRNAs encoding IL-4R, PPAR-γ, KLF4, STAT6, and SOCS1 increased significantly (*p* < 0.01).

## 3. Discussion

FLHS is a common nutritional metabolic disease of laying hens and usually occurs during the peak laying period in chickens. The disease is characterized by the presence of large numbers of fat deposits and extensive hemorrhaging in the liver [9,10]. This often leads to a sharp decline in egg production and shortening of the peak egg production period, with a mortality rate ranging from 40–70% in laying hens [11]. Some studies have shown that the development of FLHS is related to factors such as nutrition, genetic predisposition, environment, management practices, and gut microbiota [12]. The pathogenesis of FLHS is similar to that of NAFLD in humans, but details remain unclear, and effective prevention and treatment methods are lacking.

Establishing a model of FLHS in laying hens is necessary for investigating the pathogenesis of the disease and potential control measures. Various methods have been reported for developing a model of FLHS in laying hens, including exogenous injection of estradiol benzoate [13] and adjustment of dietary ratios [14,15]. However, feeding birds a high-energy, low-protein diet is currently the most common method for generating FLHS model hens because of its simplicity and ease of implementation. The pathogenesis, disease course, and onset of symptoms are highly similar in FLSH model hens to FLHS that occurs naturally in production practice.

In this study, we constructed an FLHS model by feeding laying hens a high-energy, low-protein diet. We found enlargement of the liver in FLHS model laying hens, with clear signs of hemorrhaging. In addition, the liver was brittle and friable. Histologic analysis of the liver of FLHS laying hens revealed destruction of hepatocytes, blurring of the traces of hepatic cords, and an increased number of fat vacuoles with clear signs of steatosis. Levels of ALT and AST in the liver of FLHS laying hens were significantly elevated, indicating that generation of the FLHS model in laying hens was successful. Furthermore, the disease characteristics of FLHS model hens were consistent with previous reports [16,17].

Intervention by treatment with CDCA resulted in a general restoration of the liver to normal size and color in FLHS laying hens, and bleeding was reduced. Structurally, the liver of FLHS laying hens gradually normalized after CDCA treatment, and the fatty vacuoles were reduced. Levels of ALT and AST in the liver of FLHS laying hens declined significantly after treatment, indicating that CDCA treatment significantly ameliorates the effects of liver injury in FLHS laying hens. Although there are no reports in the literature on CDCA improving FLHS in laying hens, both animal model and clinical studies have shown that artificial addition of bile acids or bile acid analogues to the diet can reverse liver injury caused by various factors [18,19,20]. Fu et al. reported that the addition of obeticholic acid to the diet ameliorated the effects of liver injury in NASH mice by reducing the rate of cell death [21]. Huang et al. showed that CDCA intervention attenuated high-fat diet–induced liver injury in rats [22]. A study by Aljarboa et al. found that addition of CDCA ameliorated valproic acid–induced liver injury in rats [23].

Several studies have demonstrated that in a state of fatty liver disease, hepatic macrophages exhibit increased polarization toward the M1 type and produce a variety of pro-inflammatory mediators. The inflammatory response induced by these pro-inflammatory factors is an important cause of liver injury in fatty liver disease [24]. M1-type macrophage markers include the scavenger receptor CD68 and iNOS, the high expression of which drive further inflammatory and immune responses [25]. Macrophages can be polarized to the M1 type following stimulation with inducers such as IFN-γ or LPS, and M1-type macrophages secrete high levels of pro-inflammatory factors such as IL-1β, TNF-α, IL-18, IL-6, and IL-8. Over-expression of these pro-inflammatory factors in the liver can directly trigger an inflammatory response, leading to liver injury. Furthermore, production of these pro-inflammatory factors induces the release of iNOS by M1-type macrophages, thereby exacerbating inflammatory injury in the liver [26]. We found that the expression levels of mRNAs encoding the hepatic M1-type macrophage markers CD68 and iNOS were significantly elevated in FLHS laying hens, as were levels of the pro-inflammatory factors IL-1β, IL-6, IL-8, IL-18, iNOS, and TNF-α, suggesting that hepatic injury in FLHS laying hens is related to the pro-inflammatory response induced by the polarization of hepatic macrophages to the M1 type.

In contrast, the mRNA and protein expression levels of these pro-inflammatory markers were significantly reduced after CDCA treatment. Levels of IL-18, iNOS, and TNF-α were reduced to the level of the control group, suggesting that CDCA ameliorates the effects of liver injury in FLHS laying hens by inhibiting the pro-inflammatory response induced by M1-type polarization of hepatic macrophages. Mohamed et al. reported that CDCA reduces inflammation in model rats fed a high-fat diet by inhibiting the secretion of pro-inflammatory factors such as TNF-α and IL-6 [27]. In a study using maternal and placental tissue samples from intrahepatic cholestasis of pregnancy, Brenøe et al. found that intervention with bile acids inhibited macrophage M1-type polarization by suppressing the expression of M1-type macrophage markers [28]. Zhuang et al. reported that knockdown of the gene encoding the bile acid receptor Gpbar-1 significantly accelerated the process of macrophage M1-type polarization and exacerbated acute liver injury, inflammation, and hepatocyte apoptosis in mice with acute cholestatic liver injury [29].

Recent studies have reported that JAK-STAT, LPS-TLR4-MyD88-NF-kB, and NLRP3 are all involved in macrophage polarization and the resulting secretion of pro-inflammatory mediators. When INF-γ acts on specific receptors (INFR) on the surface of macrophages, it activates expression of the transcription factor STAT1 by recruiting activated JAK2 protein, which initiates the expression of M1-type polarization–associated genes that promote the secretion of pro-inflammatory factors, thus intensifying the inflammatory response and consequently exacerbating damage to tissues and organs [30]. Our analyses showed that the expression levels of IFN-γ, JAK2, STAT1, and IRF5, regulators related to the JAK2-STAT1 signaling pathway, were significantly elevated in FLHS laying hens. Expression of IFN-γ, JAK2, STAT1, and IRF5 was significantly reduced after CDCA treatment, suggesting that CDCA inhibits the pro-inflammatory response mediated by M1-type macrophage polarization in the liver of FLHS laying hens via a mechanism related to down-regulation of the JAK2-STAT1 signaling pathway. Zangerolamo et al. reported that the addition of TUDCA reduced inflammatory damage in the hypothalamus in Alzheimer’s disease model mice by inhibiting the activation of factors such as p-JAK2 and p-STAT3 [31]. Attia et al. showed that obeticholic acid attenuated the onset and progression of NASH in mice by suppressing expression of proteins such as JAK2 [32]. A study by Renga et al. showed that IFN-γ–induced STAT1 dimerization resulted in transcriptional repression of the bile acid receptor FXR gene in macrophages and promoted the production of inflammation-related cytokines [33]. In a study of autoimmune hepatitis in mice, Meng et al. reported that inhibition of JAK2-STAT1 signaling pathway activation significantly suppresses IFN-γ-induced hepatic inflammatory injury resulting from apoptosis of mouse hepatocytes [34].

The inflammatory response mediated by the LPS-TLR4-MyD88-NF-κB signaling pathway also plays an important role in the pathogenesis of NAFLD. TLR4, a member of the Toll-like receptor family, is generally activated by LPS to promote an inflammatory response. This process usually facilitates the removal of exotoxins [35]; however, excessive inflammation may cause more damage than the external infection [36]. TLR4 is commonly expressed by macrophages, monocytes, and dendritic cells and binds specifically to LPS, which in turn activates downstream signaling pathways, including the MyD88-dependent pathway [37,38]. The MyD88-dependent pathway is the primary downstream pathway activated by TLR4, which first activates IL-1 receptor–associated kinase and then further binds TRAF6 to mediate the phosphorylation of IκB, ultimately leading to the nuclear translocation of various transcription factors such as NF-κB, AP-1, and IRF1 [39]. In the liver, NF-κB triggers a series of signaling cascades within M1-type macrophages via MyD88-dependent and MyD88-independent pathways, thereby stimulating the release of numerous pro-inflammatory factors that initiate an inflammatory response.

In the present study, expression of LPS was significantly elevated in FLHS laying hens, and the expression of TLR4, MyD88, NF-κB, AP-1, and IRF1 mRNAs was also significantly elevated. By contrast, the expression of factors related to the LPS-TLR4-MyD88-NF-κB pathway decreased significantly after CDCA treatment, suggesting that the mechanism by which CDCA suppresses the pro-inflammatory response mediated by M1-type polarization of macrophages in the liver of FLHS laying hens is related to inhibition of LPS-TLR4-MyD88-NF-κB signaling pathway activation. Using an LPS-induced liver injury rat model, Milivojac et al. found that CDCA treatment significantly ameliorated LPS-induced liver injury by decreasing LPS levels [40]. Dai et al. reported that treatment with CDCA reduces cancer-induced inflammation by inhibiting the LPS-TLR4-MyD88-NF-κB signaling pathway in cholangiocarcinoma cells [41]. A study by Yang et al. reported that treatment with bile acid receptor agonists ameliorated hepatic inflammation-related injury in ANIT-stimulated cholestatic hepatitis mice by decreasing LPS levels and blocking the LPS-TLR4-MyD88-NF-κB signaling pathway [42]. Mobraten and others have reported that treatment with bile acids such as CDCA inhibit activation of the NF-κB signaling pathway in U937 and human primary CD14+ monocytes, thereby suppressing the LPS-induced cellular inflammatory response [43].

In recent years, extensive research has focused on the role of inflammatory responses mediated by inflammasome NLRP3 in liver injury caused by metabolism-associated fatty liver disease. NLRP3, an important sensor in the innate immune system, detects invasion by exogenous pathogens and endogenous cellular damage and responds by forming NLRP3 inflammatory vesicles. At the onset of metabolism-associated fatty liver disease, LPS is produced in large quantities, hepatic macrophages are polarized toward the M1 type, and NLRP3 inflammatory vesicles are activated, which promotes the transcription and protein synthesis of inflammatory vesicle–associated components, ASC, Caspase-1, and Caspase-11. This in turn induces the maturation and secretion of pro-inflammatory factors such as IL-1β and IL-18, initiating an inflammatory response that ultimately produces inflammation-related injury [44]. The results of the present study showed that expression of NLRP3, Caspase-1, and Casepase-11 at the mRNA level was significantly higher in the FLHS group compared with the control group. The expression of each of these factors decreased significantly after CDCA treatment, indicating that CDCA directly inhibits the synthesis of NLRP3, thereby reducing the inflammatory response and ameliorating the effects of liver inflammation-related injury. In an in vivo study, Gong et al. reported that inhibition of NLRP3 inflammasomes by Caspase-1 inhibitors significantly reduced IL-1β levels in liver tissues of mice, thereby ameliorating the effects of liver injury induced by hepatic fibrosis [45]. Furthermore, Oleszycka et al. showed that CDCA reduces LPS-induced inflammatory damage in mouse dendritic cells by down-regulating the expression of NLRP3 and Caspase-1. A study by Haoran et al. found that treatment of mice with CDCA reduces the activity of NLRP3 inflammatory vesicles and Caspase-1, thereby inhibiting the secretion of pro-inflammatory factors such as IL-1β, IL-6, and TNF-α and attenuating stress-induced inflammatory injury [46].

Other recent NAFLD model studies have confirmed that activation of M2-type macrophages in NAFLD models leads to down-regulation of cytokine and inflammation-related gene expression, thereby reducing the extent of liver injury [47,48]. Macrophage M2-type polarization is associated with significant up-regulated expression of the markers CD163, CD206, and ARG, which leads to secretion of the anti-inflammatory factors IL-10, IL-4, IL-13, and TGF-β to exert anti-inflammatory effects. In the present study, expression levels of CD163, CD206, and ARG mRNAs and protein levels of IL-4 and IL-10 were significantly reduced in FLHS laying hens, suggesting that liver injury in FLHS laying hens is associated with anti-inflammatory responses induced by M2-type polarization of hepatic macrophages. However, CDCA treatment resulted in significantly higher expression of CD163, CD206, and ARG mRNAs and significantly higher levels of IL-4 and IL-10 protein, suggesting that CDCA ameliorates the effects of liver injury in FLHS laying hens by spiking the anti-inflammatory response induced by M2-type polarization of hepatic macrophages. A study by Grander et al. found that Nor UDCA treatment reduced ethanol-induced hepatic expression of the pro-inflammatory cytokines IL-1β and IL-6 and attenuated liver injury via a mechanism related to the promotion of hepatic macrophage polarization toward the M2 phenotype [49]. Du et al. reported that treatment with CDCA promotes macrophage polarization toward the M2 type by increasing IL-10 expression, thus attenuating high-fat diet–induced inflammatory injury in the liver and intestines of *Caenorhabditis elegans* [50]. Shao et al. reported that treatment with LCA inhibits glycolysis, promotes oxidative phosphorylation and macrophage polarization toward the M2 type, and reduces liver injury induced by hepatic fibrosis in mice [51]. Liu et al. reported that CDCA promotes macrophage polarization toward the M2 type and attenuates inflammatory damage in tissues such as the liver in mice with acute myeloid leukemia [52].

Other studies have shown that M2-type macrophage polarization can be initiated by the activation and phosphorylation of STAT6 through activation of JAK1 and JAK3 following binding of IL-4 to IL-4Ra on the cell membrane [53] and STAT6 binding to the transcription factors KLF4 and PPAR-γ [54]. M2-type macrophage polarization can also be initiated by activation of STAT3 by IL-10 through binding to IL-10R [55]. The SOCS family of signal transduction inhibitors may be involved in the regulation of macrophage polarization by negatively regulating JAK-STAT activity [56]. The results of the present study showed that expression of the IL-4R, STAT6, SOCS1, PPAR-γ, and KLF4 mRNAs was significantly reduced in FLHS laying hens. By contrast, expression of IL-4R, STAT6, SOCS1, PPAR-γ, and KLF4 mRNAs was significantly elevated after CDCA treatment, suggesting that CDCA promotes the polarization of M2-type macrophages and increases the levels of anti-inflammatory mediators in the liver of FLHS laying hens by up-regulating the mRNA expression of factors related to the JAK-STAT signaling pathway. Chen et al. demonstrated that CDCA ameliorates the effects of inflammatory liver injury induced by feeding a high-fat diet in mice by increasing PPAR-γ transcriptional activity and promoting macrophage M2-type polarization [57]. Experiments by Lv et al. showed that THDCA addition reduces drug modeling–induced inflammatory injury in mouse intestine by increasing the secretion of IL-4 and IL-10 [58]. Reports by Chen et al. showed that treatment with UDCA negatively regulates SOCS1 expression, down-regulates STAT phosphorylation, promotes macrophage polarization toward the M2 type, and ameliorates the effects of liver injury in NAFLD mice [59].

## 4. Materials and Methods

### 4.1. Experimental Design

A total of 216 Hyland Brown laying hens, 26 weeks of age (peak laying age) and of similar body weight, were selected for use in the study. The basal diet was formulated according to NRC (1998) standards. High-energy low-protein feed ratios are corn: 35–45%, millet polished meal: 5–15%, pepper seeds: 3–8%, soybean meal: 4–7%, stone pellets: 8–10%, citric acid residue: 1–3%, corn sugar residue: 1–5%, peanut cake: 1–6%, corn germ cake: 3–6%, sesame seed cake: 1–7%, soybean oil: 0.5–1%, beet molasses: 1–2%. Calcium Hydrogen Phosphate: 0.5–0.8%, 60% Choline Chloride: 0.1–0.2%, Sodium Chloride: 0.25–0.35%, Amino Acids: 0.25–0.85%, Mineral Premix: 0.2%, Vitamin Premix: 0.2%. CDCA was purchased from Hebei Huaheng Biotechnology Co, LTD, Hebei, China. The hens were housed in three-dimensional cages used on egg farms for rearing and managed according to the same practices used in farm egg operations. The temperature in the chicken house was maintained at 21–25 °C, with a relative humidity of 45–65%, and the chicken house was disinfected once per week. The hens also received all routine immunizations.

Laying hens were pre-fed for approximately 1 week, and those exhibiting only small individual differences and good body condition without signs of disease or abnormality were selected and randomly divided into six groups of three replicates each, with twelve hens in each replicate; the grouping protocol and treatments are summarized in Table 1. At the end of rearing, blood samples were collected and centrifuged to collect serum, after which the hens were sacrificed and the livers collected for subsequent analyses. All procedures used in the animal experiments were reviewed and approved prior to the study by the Animal Care and Use Committee of Shenyang Agricultural University (approval no. 2019030101).

### 4.2. Histopathological Analysis

Tissue blocks of laying hen liver samples were fixed in 4% paraformaldehyde, removed from the specimen bottles, and prepared for histopathological analysis as follows: the sections were rinsed, dehydrated, and made transparent, immersed in wax and embedded, and then sectioned and affixed. The procedures were carried out according to the instructions provided with the HE staining kit. After staining, the sections were sealed using neutral gum, dried completely, and observed under a microscope.

### 4.3. Determination of Serum Biochemical Indices

Serum samples were analyzed to determine the concentrations of ALT and AST using the colorimetric technique outlined in the instructions provided with the assay kit (Nanjing Jiancheng Bioengineering Institute, Nanjing, China).

### 4.4. Detection of Inflammatory Factors in Liver Tissue

IL-1β, IL-6, TNF-α, IFN-γ, IL-4, and IL-10 in the liver tissues of experimental laying hens in each group were analyzed using a multifactor assay. An ELISA kit (Nanjing Jiancheng Bioengineering Institute, China) was used to determine levels of IL-8, IL-18, and inducible nitric oxide synthase (iNOS) in the liver tissue of experimental laying hens.

### 4.5. Real-Time Polymerase Chain Reaction (PCR) Analysis

RNA was extracted from liver tissue using Trizol reagent, and the purity of the extracted total RNA was assessed using a microplate reader. The OD_260_/OD_280_ ratio was measured after adjusting to zero using double-distilled water. The ratio was in the range of 1.8–2.0, indicating that the purity of the RNA was sufficient for the experimental requirements. The RNA was then converted into cDNA using a reverse transcription kit according to the manufacturer’s instructions.

mRNA expression levels of related factors were measured using real-time quantitative PCR. The full sequences of the genes were searched in GenBank on NCBI, and Primer Premier5 was used to design the primer sequences, which are listed in Table 2. After primer specificity was verified as sufficient, SYBR Green II was used for real-time quantitative PCR, using β-actin as an internal reference to normalize target gene expression. The mRNA data were analyzed using the 2^−ΔΔCT^ method.

### 4.6. Statistical Analysis

The resulting data were analyzed using SPSS 23.0 software. The significance of differences between groups was evaluated using one-way analysis of variance. Multiple comparisons were performed using the LSD method. Data are expressed as the mean and standard error of the mean (SEM). *p* < 0.05 was defined as indicating statistical significance.

## 5. Conclusions

The results of this study showed that prophylactic administration of CDCA significantly ameliorates the effects of liver injury in FLHS laying hens by inhibiting macrophage M1-type polarization and the resulting pro-inflammatory response via down-regulation of the JAK-STAT, LPS-TLR4-MyD88-NF-κB, and NLRP3 pathways and up-regulation of the JAK-STAT pathway to promote macrophage M2-type polarization and the resulting anti-inflammatory response. These results provide new insights that could aid efforts to elucidate details regarding the pathogenesis of FLHS in laying hens and facilitate the use of CDCA to prevent FLHS.

## Figures and Tables

**Table 1:** Animal groups and treatments.

Groups	*n* Values	Treatments
Normal group (C)	n = 36	Corn-soya-meal–based feed
FLHS model group (M)	n = 36	High-energy, low-protein feed
CDCA low-dose group (CDCA L)	n = 36	High-energy, low-protein feed + 0.01% CDCA
CDCA medium-dose group (CDCA M)	n = 36	High-energy, low-protein feed + 0.02% CDCA
CDCA high-dose group (CDCA H)	n = 36	High-energy, low-protein feed + 0.03% CDCA
C+ CDCA (CC)	n = 36	Corn-soya-meal–based feed + 0.02% CDCA

**Table 2:** Primer sequences used for fluorescence quantitative real-time PCR.

Gene	Primer Sequences		ID
iNOS	F: ATCTACAGGTATTGATGCTCGT	R: TTCTGGATCTTGGCCGTTTG	35671
JAK2	F: CTGGCTTCTACGTTCTTCGT	R: GGAGGTTTGATTTATCTTTTGG	374199
STAT1	F: TACTTATGACCCTGACCCTATC	R: TTTCCTGAATCCTTTGACTG	424044
IRF5	F: ATCCAAGTGTTCAGCCTCCA	R: CTCCACCAGAGCATCCTTCA	430409
TLR4	F: GGAGGTTGTAGATTTGATGAG	R: AGATGGGACATAACATGATTT	417241
MyD88	F: GAGTTGGAGCAAACGGAGTT	R: TTGGTGCAAGGATTGGTGTA	420420
NF-κB	F: AGGTGGTCCCTAAGTTCCGTG	R: TTTGCCTCTTGGTGCGTTTC	396093
AP-1	F: AACTTCGTGCCCACCGTGAC	R: CCGCTGCCATCTTGTTCCTC	102587711
IRF1	F: AGCATTGAGGATATCGTGAAG	R: TGGTTGTGGTCTGTGCTGTGT	396384
NLRP3	F:AAAGGACGTGAATATGTTGTTA	R: CAAGGCTATTCCTGTGAAACT	423021
Caspase-1	F: GCTGCCGTGGAGACAACATA	R: CGTTGGACCTTTCGGAACAT	395764
Caspase-11	F: AGTCAGAGCACAGGACGAAG	R: GATGGGAAGAGGAAGAGAG	395476
CD163	F: TGGTTCCGCTCATTTTGGTC	R: GGGCAGTTTCAGTTCCTTTAC	426826
CD206	F: GCATCAAGCGTATTTAGCAA	R:GAAAGTCCAATCCAAAAGTAT	9332
ARG	F: TGATCTTGGAGTCATCTGGG	R:GTCCGTCAACATCAAAACTTAG	46717
IL-4R	F: CAGCATCACCAAGATTAGAA	R: TCCAGAAAACAGGGCAAGAG	3566
STAT6	F: CTGGGAGAAGATGTGCGATAC	R: TTGCTGATGAAGCCAATGAT	100859196
SOCS1	F: CGATGTCTACTTGACCCTCC	R: CCCCGTCTGAAAGTTTATCC	416630
PPARy	F: GCAGGAACAGAACAAAGAAG	R: TGCCAGGTCACTGTCATCTA	100356422
KLF4	F: CCTTCAACCTGGCGGACATC	R: CTGGCCTCCTGCTTGATTTT	770254
β-Actin	F: GGAGGGAAATCGTGCGTGACA	R: CGATAGTGACCTGACCGTCA	396526

## Data Availability

The data used to support the findings of this study are available upon request from the corresponding author.
